# Animal shelter management of feline leukemia virus and feline immunodeficiency virus infections in cats

**DOI:** 10.3389/fvets.2022.1003388

**Published:** 2023-01-18

**Authors:** Paola Dezubiria, E. Susan Amirian, Keegan Spera, P. Cynda Crawford, Julie K. Levy

**Affiliations:** ^1^Maddie's Shelter Medicine Program, College of Veterinary Medicine, University of Florida, Gainesville, FL, United States; ^2^Research Department, Austin Pets Alive!, Austin, TX, United States

**Keywords:** feline leukemia virus, feline immunodeficiency virus, animal shelter, cats, diagnostic testing, euthanasia, pet adoptions, Shelter Medicine

## Abstract

Approximately 5% of cats in animal shelters in the United States test positive for either feline leukemia virus (FeLV) or feline immunodeficiency virus (FIV), which translates to more than 100,000 positive cats managed by shelters each year. Little is known about the current status of retroviral management in animal shelters, particularly in regions burdened by chronic pet overpopulation and high shelter admissions, such as the southern United States. The purpose of this study was to describe feline retroviral management in Florida shelters. Shelters were surveyed on practices including selection of cats for testing, diagnostic techniques, and outcome options for cats with positive test results. Responses were received from 139 of 153 animal shelters known to admit cats, including 55 municipal shelters (40%), 70 private shelters (50%), and 14 private shelters with municipal contracts (10%). A total of 115 shelters (83%) performed at least some testing, most using combination point-of-care devices for simultaneous FeLV antigen and FIV antibody screening. Of shelters that performed any testing, 56 (49%) tested all cats for FeLV and 52 (45%) tested all cats for both FeLV and FIV. The most common reason for testing was screening adoptable cats (108 shelters; 94%) and cats available for transfer to other organizations (78; 68%). Testing cats in trap-neuter-return/return-to-field programs was least common (21; 18%). Most common outcome options for positive cats included adoption (74; 64%), transfer (62; 54%), and euthanasia (49; 43%). Euthanasia following a positive test result was more common for cats with FeLV (49; 43%) than for cats with FIV (29; 25%) and was more common in municipal shelters, rural shelters, shelters taking in <500 cats a year, and shelters with overall live outcome rates for cats <70%. Although Florida shelter compliance with national guidelines for identification and management of FeLV and FIV positive cats was variable, most had live outcome options for at least some of their cats with positive test results. Increased access to training and practical programmatic tools may help more shelters implement cost-effective testing protocols, reduce risk for transmission to other cats, and support the best outcomes for this vulnerable population of cats.

## Introduction

Feline leukemia virus (FeLV) and feline immunodeficiency virus (FIV) are retroviruses that are among the most common infectious diseases of cats (1). FeLV is spread by close contact among cats, especially from queens to kittens. Cats with regressive FeLV infection associated with a robust anti-viral immune response have low viral loads, minimal infectious virus shedding, lack of clinical signs, and prolonged survival, whereas cats with progressive FeLV infection have high viral load, infectious viral shedding in secretions, and increased risk of clinical disease and premature mortality within a few years of diagnosis (2). In contrast, cats with FIV are most commonly infected as adults *via* bite wounds and often experience a prolonged asymptomatic phase with many living normal lifespans.

The American of Association Feline Practitioners (3) and the Association of Shelter Veterinarians (4) advise against euthanasia of cats solely on the basis of retroviral infection and have established guidelines for the care, adoption, and post-adoption management of FeLV and FIV infected cats in shelters (3, 4). The AAFP generally recommends that all cats, with the exception of those in trap-neuter-return programs, be tested for FeLV and FIV at the time of acquisition. The AAFP further recommends screening shelter cats for FeLV prior to mingling in multi-cat community rooms, whereas screening cats housed alone in single-cat enclosures can be deferred until after adoption when families select their veterinarians for ongoing care. Because FeLV can be spread among cats that co-habitate, it is recommended that FeLV-infected cats be housed alone or with other FeLV-infected cats. In contrast, transmission of FIV among co-habituating cats is uncommon unless they fight, so there is less certainty about the need to segregate FIV-infected cats from uninfected cats as long as they are amicable (5). An exception to the universal testing guideline exists for healthy free-roaming community cats enrolled in trap-neuter-return (TNR) and return-to-field (RTF) programs (1, 3). Most testing is performed in shelters and veterinary clinics using blood samples in rapid point-of-care devices. In the United States, devices can be selected that test for FeLV alone or for both FeLV and FIV simultaneously, but not for FIV alone. Samples may also be submitted to reference laboratories for more extensive testing.

Approximately 5% of cats in US shelters test positive for either FeLV or FIV (6), which translates to more than 100,000 positive cats managed by shelters each year based on an estimated shelter cat intake of 2.2 million (7). Increasingly, shelter programs aim to save all animals that are not suffering or dangerous to society, even if their long-term outcome is uncertain (7). As a result, some shelters have developed adoption programs for FeLV and FIV positive cats (1, 3, 8). Little is known about the current status of retroviral management in animal shelters, particularly in regions burdened by chronic pet overpopulation and high animal shelter admissions, such as the southern United States. While euthanasia rates due to shelter crowding and disease have decreased over time, cats are still twice as likely to be euthanized in shelters compared to dogs (7). The purpose of this study was to determine how animal shelters in Florida manage retroviral infections of cats, including selection of cats for testing, diagnostic techniques, and outcome options for cats with positive test results.

## Materials and methods

### Animal shelters

For the purpose of this study, an animal shelter was defined as a continuously occupied “brick and mortar” physical facility that housed cats and/or dogs temporarily for animal control and/or animal protection in the state of Florida. Common examples included shelters operated by municipalities and humane societies. Foster-based animal rescue organizations and sanctuaries with a permanent animal population were not included in the study. Shelter types included municipal (operated by a town or county), private (operated by a non-profit or business entity), or private with municipal contract (holding a contract to provide animal control and/or sheltering services for a municipality). Counties of shelter location were defined as rural (<100 residents per square mile) or urban (≥100 residents per square mile). Since the State of Florida does not maintain a registry of animal shelters in Florida, the Shelter Medicine Program at the University of Florida has maintained an annually updated directory and state-wide shelter-level statistics on cat and dog admission and outcome data since 2013. These previously collected data for 2019 were used to calculate live outcome rate as the number of cats released alive in 2019 divided by the number of live cats taken in during 2019.

### Data collection

A prototype survey regarding selection of cats for testing, testing protocols, and outcomes for cats with positive test results was administered to a focus group of six shelter representatives to assess for clarity and ease of use. Their feedback was used to create a final survey instrument comprised of 15 questions regarding FeLV and FIV practices (Supplemental material—Survey Form). Survey respondents were instructed to describe “the most common protocols that were routinely followed, not exceptions or unusual circumstances.” Contact information of the respondent was collected to enable follow-up clarification of any incomplete or internally inconsistent responses. Therefore, the survey was not anonymous. No individual cat-level data were collected. Data were entered into a secure GoogleSheets spreadsheet using a university-owned account.

The survey was conducted between June and September, 2020. Initial distribution was by email, with options to reply *via* multiple methods to account for variability in shelter communications policies and access. The survey could be completed online *via* a GoogleForms link, by email, fax, phone interview, or postal mail. Respondents were instructed to report practices related to management of FeLV and FIV in place during 2019, prior to the impact of lockdowns and staffing shortages related to the COVID pandemic. Research staff assisted with the collection of data from respondents without internet access and from those indicating a preference for telephone communication by reading the survey to them. Reminders were emailed to non-responders every 3 weeks beginning at Week 2. Telephone calls were attempted to non-responders every 3 weeks beginning at Week 3. A paper copy of the survey with return stamped envelope was sent by postal mail to non-responders at Week 4.

### Statistical analysis

Frequencies were used to examine proportions of shelters performing diagnostic testing for FeLV or FIV, the selection of test types, follow-up testing for positive screening tests, and outcomes available for cats with positive test results. Percentages were calculated for categorical variables, using appropriate denominators (e.g., the number of complete responses for each question). FeLV and FIV management practices were examined in relation to shelter type, shelter region, and shelter cat intake and outcome numbers.

## Results

### Animal shelter characteristics

Responses were received from 139 of the 153 animal shelters (91%) known to be taking in cats in Florida. Respondents included 55 municipal shelters (40%), 70 private non-profit shelters (50%), and 14 private shelters (13 non-profit and 1 for profit) with municipal contracts (10%). This list was developed and used by the research team for shelter data tracking since 2013. These Florida shelters collectively admitted 213,060 cats in 2019, which represented 99% of the 215,386 cats known to be admitted to shelters across the state. Most of the cats in surveyed shelters were classified as free-roaming strays (139,555; 66%), followed by surrendered pets (42,664; 20%), and other intake reasons (30,841; 14%). The shelters reported live outcomes for 162,630 cats (76%) through adoptions (101,317; 48%), transfers to other organizations (26,781; 13%), return-to-field (27,601; 13%), return to owner (4,110; 2%), and other outcomes (2,821; 1%).

### Selection of cats for testing

A total of 115 shelters (83%) performed at least some testing for FeLV and/or FIV (Table 1). Most testing shelters screened cats for both FeLV and FIV, but three tested only for FeLV. Of the 115 shelters that performed any testing, only 56 (49%) tested all cats in their care for FeLV and only 52 (45%) tested all cats for FIV (Table 1). The population most commonly tested was cats available for adoption; 108 shelters (94%) tested at least some of their adoptable cats (Table 1). A total of 78 shelters (68%) tested at least some cats available for transfer to other organizations. The population least commonly tested was cats for TNR/RTF programs; only 10 (16%) of the 61 shelters that participate in these programs tested at least some cats. FIV and FeLV testing practices were similar regardless of housing types (individual enclosures vs. group housing) and underlying health status of cats.

**Table 1:** Proportion and characteristics of cats routinely tested for FeLV and FIV and testing procedures used in 139 Florida animal shelters.

	**FeLV**		**FIV**	
	**No. of shelters**	**Percent**	**No. of shelters**	**Percent**
**Proportion of cats tested**
All cats	56	40%	52	37%
Some cats	59	42%	60	43%
No cats	24	17%	27	19%
Total	139	100%	139	100%
Types of cats tested^*^
Cats for adoption	108	94%	101	90%
Cats for transfer to other organizations	78	68%	75	67%
Cats for trap-neuter-return or return-to field^**^	10	16%	8	13%
Cats housed individually in the shelter	94	82%	89	79%
Cats housed in group housing in the shelter	93	81%	85	76%
Cats that are sick or injured	98	98%	96	86%
Test used for routine screening^*^
IDEXX SNAP^®^ point-of-care	70	61%	67	60%
Zoetis WITNESS^®^ point-of-care	23	20%	21	19%
Zoetis VETSCAN^®^ point-of-care	15	13%	15	13%
Commercial diagnostic laboratory	0	0%	0	0%
Don't know/other	11	10%	11	10%
Follow-up procedures for positive screening test results^*^
Follow-up tests are not routinely performed	72	63%	75	67%
Repeat original test	9	8%	12	11%
IFA test	11	10%	NA	NA
PCR test	9	8%	3	3%
Antigen test at laboratory	7	6%	NA	NA
Antibody test at laboratory	NA	NA	1	1%
Western blot	NA	NA	4	4%
Don't know/other	14	12%	14	13%

* More than one response could be selected.

* Proportions are calculated based on 115 shelters that tested for FeLV and 112 shelters that tested for FIV.

** Proportions are based on 61 shelters with trap-neuter-return or return-to-field programs.

NA, not applicable, this type of test is not routinely available for the indicated virus.

### Selection of tests for FeLV and FIV

Among the shelters that tested cats for FeLV and FIV, point-of-care tests that screen blood simultaneously for both FeLV antigen and FIV antibody were used most commonly (Table 1). Most shelters did not follow-up with confirmatory testing procedures following an initial positive screening test.

### Relationship of shelter characteristics with testing patterns

Of 139 shelters that participated in the study, 81 (58%) tested all cats other than those in TNR/RTF programs for FeLV, and 63 shelters (45%) also tested all cats for FIV using combination tests (Table 2). Universal testing of cats was most common in private shelters, followed by private shelters with municipal contracts, and lowest for municipal shelters. Universal testing was also more common in shelters located in urban counties than in rural counties. The smallest shelters, those taking in <500 cats in 2019, and those with live outcomes rates of <70% were more likely to forego testing altogether compared to larger shelters and those with higher live outcome rates.

**Table 2:** Relationship between shelter characteristics and FeLV and FIV testing patterns of shelter cats in 139 Florida shelters.

	**No. shelters**	**Test all cats**		**Test some cats**		**Test no cats**	
		**FeLV**	**FIV**	**FeLV**	**FIV**	**FeLV**	**FIV**
**Shelter type**
Municipal shelter	55	19 (34%)	9 (16%)	18 (32%)	27 (48%)	19 (34%)	19 (35%)
Private shelter + contract	14	7 (50%)	5 (36%)	4 (29%)	6 (43%)	3 (21%)	3 (21%)
Private shelter	70	55 (79%)	49 (70%)	12 (17%)	16 (23%)	3 (4%)	5 (7%)
Total	139	81 (58%)	63 (45%)	34 (24%)	49 (35%)	24 (17%)	27 (19%)
**Shelter region**
Rural county	31	9 (29%)	8 (26%)	9 (29%)	9 (29%)	13 (42%)	14 (45%)
Urban county	108	72 (67%)	55 (51%)	25 (23%)	40 (37%)	11 (10%)	13 (12%)
Total	139	81 (58%)	63 (45%)	34 (24%)	49 (35%)	24 (17%)	27 (19%)
Shelter cat intake^*^
1–499	57	30 (53%)	28 (49%)	9 (16%)	9 (16%)	18 (32%)	20 (35%)
500–1,499	34	21 (62%)	17 (50%)	10 (29%)	14 (41%)	3 (9%)	3 (9%)
1,500–2,999	27	17 (63%)	12 (44%)	9 (33%)	14 (52%)	1 (4%)	1 (4%)
3,000 and above	19	12 (63%)	5 (26%)	6 (32%)	12 (63%)	1 (5%)	2 (11%)
Total	137	80 (58%)	62 (45%)	34 (25%)	49 (36%)	23 (17%)	26 (19%)
Live outcome rate^*^
0–69%	35	12 (34%)	11 (31%)	12 (34%)	13 (37%)	11 (31%)	11 (31%)
70–89%	39	27 (69%)	14 (36%)	8 (21%)	20 (51%)	4 (10%)	5 (13%)
90–100%	63	41 (65%)	37 (59%)	14 (22%)	16 (25%)	8 (13%)	10 (16%)
Total	137	80 (58%)	62 (45%)	34 (25%)	49 (36%)	23 (17%)	26 (19%)

* The intake and live outcome rate were not available for two shelters.

Due to the uncommon use of testing in trap-neuter-return and return-to-field programs, this category was excluded from the calculations in this table.

A total of 112 shelters used devices that tested for both FeLV and FIV simultaneously, and 3 shelters used devices that tested only for FeLV.

### Outcomes of cats testing positive for FeLV or FIV

**Figure 1:**
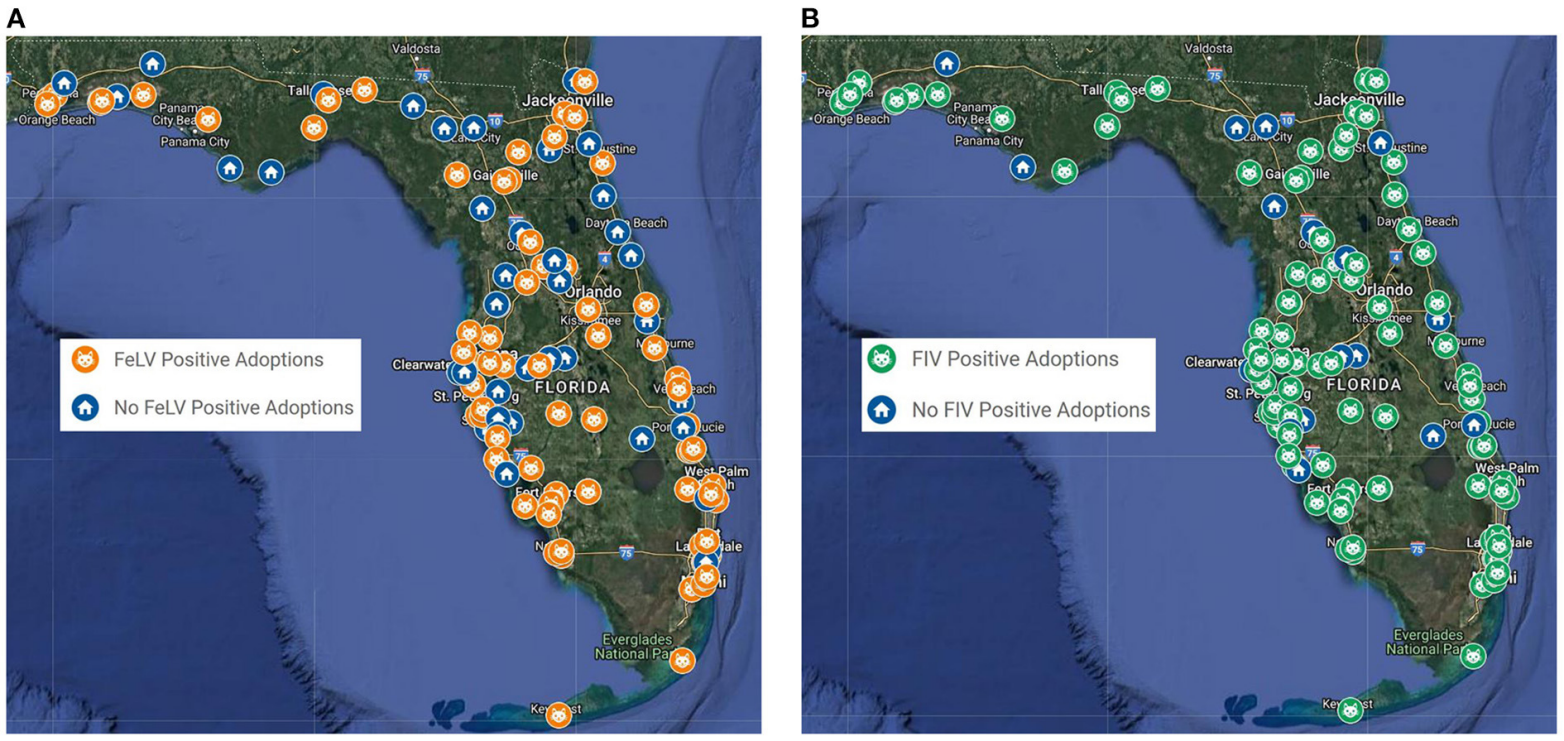
Distribution of animal shelters with adoption programs for FeLV+ cats (*n* = 74) **(A)** and FIV+ cats (*n*=93) **(B)** among 139 Florida animal shelters that took in cats.

Shelters reported multiple possible outcomes for cats with positive screening tests results (Table 3). Adoption was offered by a majority of shelters, regardless of shelter characteristics (Figure 1). In the 115 shelters that tested at least some cats for FeLV, which is more easily transmitted between cats than FIV, 63 shelters (55%) would adopt FeLV+ cats to homes with no other cats or with other FeLV+ cats, and only 11 (10%) would place cats regardless of the other cats in the homes. In the 112 shelters that tested at least some cats for FIV, which is associated with a better long-term prognosis and is rarely transmitted among compatible sterilized cats in the home environment, 38 shelters (34%) would adopt cats to homes with no other cats or with other FIV+ cats, and 55 shelters (49%) would place cats regardless of the other cats in the homes. Making positive cats available for transfer to other organizations was the second most commonly offered outcome in the 115 shelters that tested at least some cats, with 62 shelters (54%) participating in transfer programs. At total of 55 shelters (48%) transferred cats to rescue partners for eventual adoption, and 40 shelters (35%) transferred cats to sanctuaries to live out their lives. Only seven shelters would potentially return cats positive for FeLV to their neighborhoods via trap-neuter-return or return-to-field programs, although none of these shelters reported routinely testing cats for FeLV. In contrast, 23 shelters would potentially return cats positive for FIV, even though only two of those reported routinely testing for FIV. Routine euthanasia was used by 49 shelters (43%) for cats testing positive for FeLV and by 29 shelters (26%) for cats testing positive for FIV. Euthanasia was most commonly practiced in municipal shelters, rural shelters, and the shelters that admitted 3,000 cats or more in 2019. The proportion of shelters utilizing routine euthanasia for cats testing positive for FeLV and FIV was inversely proportional to the overall live outcome rate for all cats in the shelters. In all comparisons, the proportion of shelters euthanizing cats for FeLV was higher than for FIV.

**Table 3:** Relationship between shelter characteristics and the most common outcomes of cats testing positive in Florida shelters that routinely test for FeLV alone (*n* = 3) or both FeLV and FIV (*n* = 112) infection.

	**No. shelters testing**		**Adoption alone or with other positive cat(s)**		**Adopt to any home**		**Transfer to rescue/sanctuary**		**Euthanasia**	
	**FeLV**	**FIV**	**FeLV+**	**FIV+**	**FeLV+**	**FIV+**	**FeLV+**	**FIV+**	**FeLV+**	**FIV+**
**Shelter type**
Municipal shelter	37	36	14 (38%)	7 (19%)	7 (19%)	20 (56%)	26 (70%)	27 (75%)	25 (68%)	19 (53%)
Private shelter + contract	11	11	5 (45%)	3 (27%)	0 (0%)	5 (45%)	8 (73%)	9 (82%)	5 (45%)	2 (18%)
Private shelter	67	65	44 (66%)	28 (43%)	4 (6%)	30 (46%)	28 (42%)	26 (40%)	19 (28%)	8 (12%)
Total	115	112	63 (55%)	38 (34%)	11 (10%)	55 (49%)	62 (54%)	62 (55%)	49 (43%)	29 (26%)
**Shelter region**
Rural county	18	17	8 (44%)	9 (53%)	1 (6%)	1 (6%)	9 (50%)	9 (53%)	12 (67%)	11 (65%)
Urban county	97	95	55 (57%)	29 (31%)	10 (10%)	54 (57%)	53 (55%)	53 (56%)	37 (38%)	18 (19%)
Total	115	112	63 (55%)	38 (34%)	11 (10%)	55 (49%)	62 (54%)	62 (55%)	49 (43%)	29 (26%)
Shelter cat intake^*^
1–499	39	37	26 (67%)	19 (51%)	2 (5%)	14 (37%)	12 (31%)	11 (32%)	12 (28%)	5 (14%)
500–1,499	31	31	15 (48%)	9 (29%)	2 (6%)	13 (42%)	16 (52%)	15 (48%)	13 (42%)	9 (29%)
1,500–2,999	26	26	14 (54%)	7 (27%)	1 (4%)	15 (58%)	18 (69%)	20 (77%)	15 (58%)	7 (27%)
3,000 and above	18	17	7 (39%)	2 (12%)	6 (33%)	13 (76%)	15 (83%)	14 (82%)	10 (56%)	8 (47%)
Total	114	111	62 (54%)	37 (33%)	11 (10%)	55 (49%)	61 (54%)	61 (55%)	49 (43%)	29 (26%)
Live outcome rate^*^
0–69%	24	24	8 (33%)	6 (25%)	3 (13%)	8 (33%)	15 (63%)	14 (58%)	17 (71%)	11 (46%)
70–89%	35	34	18 (51%)	11 (32%)	6 (17%)	18 (53%)	22 (63%)	23 (68%)	19 (54%)	12 (35%)
90–100%	55	53	36 (65%)	20 (38%)	2 (4%)	29 (55%)	24 (44%)	24 (45%)	13 (24%)	6 (11%)
Total	114	111	62 (54%)	37 (33%)	11 (10%)	55 (50%)	61 (54%)	61 (55%)	49 (43%)	29 (26%)

* The intake and live outcome rate were not available for one shelter that tested at least some cats.

More than one response could be selected.

## Discussion

In this survey of Florida animal shelters, a majority screened at least some of the cats in their care for FeLV and FIV infection. More shelters indicated they tested cats selected for adoption programs or transfer to other organizations than for cats in TNR/RTF programs. The shelters least likely to test any cats were those with low shelter cat intake, located in rural counties, operated by municipalities, and with the lowest live outcome rates, suggesting that resource availability may underlie at least some retroviral management protocols. The AAFP defines retroviral testing in shelters as optional for individually housed cats, recommended for group-housed cats, and not recommended for cats in TNR programs (3).

Most shelters offered lifesaving options for cats with positive screening test results. Similar to patterns for testing, shelters that routinely euthanized positive cats were more likely to be low-intake, rural, municipal, and low live outcome. Shelters were more likely to offer live outcome options and less likely to routinely euthanize cats for FIV than for cats with FeLV. This is likely due to the fact that cats with FIV often experience many years of good health, may have a normal lifespan (9–11) and are unlikely to spread infection if they do not fight (5). Cats with FeLV have a shorter lifespan on average, particularly if they have a high viral load as determined by PCR at the time of diagnosis (2). However, some FeLV positive cats defy the odds and live for many years, making it impossible to predict the outcome for any individual cat. The AAFP recommends that cats positive for FeLV or FIV be offered the same life-saving options as uninfected cats, including being made available for interactions with the public in shelter adoption rooms, adoption events, and at satellite adoption centers such as pet stores as long as they are individually housed and accompanied by appropriate documentation and education (3). AAFP further recommends against regulations that prohibit interstate transport or adoption of positive cats as such restrictions are not supported by medical evidence. Most prohibitions against adoption of positive cats have been removed over time in accordance with AAFP's international guidelines for management of cats with FeLV and FIV, however the state of Kansas only began permitting adoption of cats with FIV starting in 2019 and still prohibits adoption of cats with FeLV at the time of this report.

Shelters were more likely to offer adoption to any type of household for cats with FIV, but to restrict adoption of FeLV positive cats to catless households or those with other FeLV positive cats. This indicates that policy-makers in many shelters are familiar with discoveries that transmission of FIV among compatible cats is negligible, and that positive cats can co-habitate with uninfected cats for many years without transmission (5). FeLV is more transmissible among cats with prolonged close contact. However, adult cats have a degree of natural immunity, vaccines are available to boost natural immunity, and the risk of transmission within household remains undefined (1, 3).

Making cats with FeLV and FIV available for transfer to cat rescue organizations for subsequent adoption or to sanctuaries to live out their lives was the second most common outcome opportunity routinely used by Florida shelters. Transfer programs increase the capacity for live outcomes when the original shelter has barriers such as limited space, staffing, expertise, or funds. However, processing cats into one facility and then transferring them to others may also add duplication of effort, more handling stress for the cats, and delays in reaching a final adoptive home. Whenever possible, programs should be developed that manage all cats, including those with FeLV and FIV, as efficiently through the local sheltering system in as few days as possible (12, 13) to reduce stress on cats and shelters alike. Shelters can take it one step further by supporting community-centric programs that bypass shelter intake altogether by aiding cat owners in rehoming cats themselves (14) and promoting adoption of free-roaming homeless cats directly from neighborhoods into new homes (15). Options to transfer cats to sanctuaries, which are facilities that keep animals for life, were routinely used by approximately one third of shelters. While providing for immediate live outcomes for FeLV and FIV positive cats, sanctuaries often house many cats in large groups. High-density housing can lead to stress and the spread of infectious diseases, a particular concern in cats that are already predisposed to immunodeficiency (16). Long-term survival of cats with FeLV (17) and FIV (11) is greater in low-stress homelike environments with few other cats than in high-density sanctuaries.

Only 21 shelters tested cats in TNR or RTF programs for FeLV and/or FIV, a low level consistent with many such programs in the US (18). TNR and RTF are cat population management tactics in which unowned free-roaming “community cats” are captured, surgically sterilized, vaccinated, and then returned to their neighborhoods free to live out their lives but unable to reproduce. TNR refers to capturing community cats specifically for the purpose of sterilization followed by return to their neighborhoods. RTF refers to cats originally admitted to shelters and then, after assessment, designated for sterilization and return. The only difference is the original intent of the person initiating the intervention—whether it was for the purpose of sterilization and return from the outset (TNR) or for the purpose of shelter intake (RTF). The effectiveness of TNR/RTF programs in controlling free-roaming cat populations and their impact on cat welfare, the environment, public health, and neighborhood wellbeing is directly related to the intensity in which they are delivered (19–21). If sufficiently aggressive and targeted, such programs can reduce cat numbers; if they are too sporadic, they lose their population-level impact. Sterilization also provides the benefit of reducing the primary factors in transmission of FeLV (dams infecting their kittens) and FIV (bite wounds among brawling intact male cats). The AAFP does not recommend routine testing of cats in TNR/RTF programs because cat population management, prevention of excess kitten births, individual cat welfare, and the spread of FeLV and FIV are all better served by investing available resources in sterilizing the most cats possible (1, 3).

Multiple national and local initiatives have succeeded in reducing the number of animals admitted to and dying in animal shelters. In 2019, shelter intake in the US was estimated at 2.7 million dogs and cats, of which 2.2 million were released alive (81%) (7). However, cats were half as likely to have live outcomes compared to dogs. As shelters progress in saving high proportions of healthy and well-socialized animals, increasing attention has turned to development of programs to address the specific needs of the most vulnerable and at-risk animals, including neonatal, geriatric, fearful, injured, abused, with bite histories, transient contagious diseases, and chronic manageable conditions such as FeLV and FIV (22).

A previous study indicated such a high national demand among shelters and rescue groups for placement options for their FeLV and FIV positive cats that hundreds of cats were sent each year from across the county to a specialized adoption program in Austin, Texas (8). Of 801 cats referred to the FeLV adoption program over 2 years, 19% were deemed not to be infected upon further testing, illustrating the risk of deciding a cat's fate based on a single test. Of the remaining cats, 82% were adopted, and 17% died or were euthanized. Post-adoption survey data gathered by the shelter reflected high adopter satisfaction (95% positive experience) and a low return rate (4%) for cats in the FeLV adoption program (23). The Austin program now serves as a model for shelters around the country seeking to establish their own adoption programs by providing shelter protocols, adoption marketing tips, educational materials for adopters and local veterinarians, and materials for post-adoption support.

This report had several limitations, primarily related to shelter record-keeping systems that do not allow for recovery of quantitative data on the number of cats tested, the testing results, and the outcomes of individual cats with positive test results. In addition, shelter staff often incorporate a variety of both standardized and intangible influences on protocol development and compliance that might be better surfaced in a qualitative interview-based study. These unmeasured factors may include cat-specific variables such as behavioral and health issues that may impact assumptions about an individual cat's tractability for handling or the likelihood of a positive outcome in the shelter, and shelter-specific variables, such as seasonal shelter crowding, staff availability, or budget limitations. An additional factor is the combination of conditions that individually might result in one option, but in combination with other conditions might result in a different option. As a result, it was not always clear in this study what practices were truly “routine” and what were based on situational and often multifactorial conditions.

## Conclusions

Florida shelter compliance with national guidelines for identification and management of cats testing positive for FeLV and FIV was variable. Nevertheless, most Florida shelters screened at least some cats for infection and had live outcome options for at least some of their cats with positive test results. This indicates a need to support increased knowledge transfer, protocol development, and access to practical programmatic tools for shelter decision makers to implement cost-effective testing protocols, risk reduction for transmission to other cats, enhanced adoption programs, and improved handoff of care from the shelter, where cats are rescued and then adopted, to the adopters' new veterinarians, where lifelong care will be provided.

## Data availability statement

The raw data supporting the conclusions of this article will be made available by the authors, without undue reservation.

## Author contributions

PD solicited participation in the study, collected shelter testing data, constructed the data tables, and drafted the manuscript. KS collected data on total shelter cat intake and outcomes. PC assisted in data collection and manuscript preparation. JL and EA designed the study and edited the manuscript. All authors contributed to the article and approved the submitted version.
