# Supplementary material for: Animal shelter management of feline leukemia virus and feline immunodeficiency virus infections in cats
Source: Front Vet Sci. 2023 Jan 18;9:1003388. doi: 10.3389/fvets.2022.1003388 (PMC9890067; doi:10.3389/fvets.2022.1003388)
Supplement: Supplementary file 1 [file Data_Sheet_1.PDF]

UF | Maddie's® Shelter Medicine Program
College of Veterinary Medicine
UNIVERSITY of FLORIDA

Florida Animal Shelter Survey: Management of Cats with FeLV and FIV

Thank you for completing this brief survey regarding your shelter's protocols for feline leukemia virus (**FeLV**) and feline immunodeficiency virus (**FIV**). Your data will be combined with data from other shelters for our report–your individual data will not be shared.

This survey pertains to your shelter's FeLV and FIV protocols in **2019.** This will enable us to correlate responses with 2019 statewide shelter data.

When answering the questions, please respond with the most common protocols that were routinely followed, not exceptions or unusual circumstances. This survey only applies to cats that were cared for by your shelter, not to cats that may have received care through a public clinic, if your shelter has one.

We are aiming for a complete statewide survey of Florida shelters, so would be grateful if you can submit it by **Thursday July 9, 2020**. Please do not hesitate to reach out if you have any questions.

Thank you for taking time to contribute to this study. We know how busy you are with shelter operations during normal times and that the COVID pandemic has made everything even more demanding.

Paola Dezubiria
Veterinary Student
[redacted] redacted

Dr. Julie Levy
Professor of Shelter Medicine
levyjk@ufl.edu

Five ways to submit:

- Online survey: https://bit.ly/FeLV-FIV
- Email: [redacted] redacted
- Fax: (352) 392-6125
- Phone interview: [redacted] redacted
- Mail: Maddie's Shelter Medicine Program
  College of Veterinary Medicine
  2015 SW 16th Avenue
  Gainesville, FL 32608

Start Survey

1. Shelter Name:

2. Primary County:

3. Organization Type **(choose 1)**:

   ◯ Municipal shelter
   ◯ Private shelter
   ◯ Private shelter with municipal contract

FeLV Testing

Please respond with the most common protocols that were routinely followed in 2019, not exceptions or unusual circumstances.

4. In regards to routine **FeLV** testing protocols in place in 2019 **(choose 1)**:

   ◯ **All cats** were tested **- PLEASE CONTINUE** to complete the following questions
   ◯ **Some cats** were tested **- PLEASE CONTINUE** to complete the following questions
   ◯ **No cats** were tested – **PLEASE SKIP** to the next section **FIV Testing**

5. What types of cats were routinely tested for **FeLV** in 2019? **(check at least one box in each row)**

**Type of Cat**	**No cats**	**Kittens (under 6 months old)**	**Adults (6 months & older)**	**N/A (shelter does not do this activity)**
Cats for adoption	☐	☐	☐	☐
Cats for transfer to other groups	☐	☐	☐	☐
Cats for Trap-Neuter-Return or Return-to Field	☐	☐	☐	☐
Cats housed individually in the shelter	☐	☐	☐	☐
Cats housed in group housing in the shelter	☐	☐	☐	☐
Cats that are sick or injured	☐	☐	☐	☐
Cats for whom the adopter/caregiver requests to be tested	☐	☐	☐	☐

6. If applicable and could not be answered in previous question, describe OTHER types of cats that were routinely tested for FeLV in 2019:

7. What brand of test was routinely used for the initial **FeLV** screening in 2019? **(check all that apply)**

- ☐ IDEXX SNAP
- ☐ Zoetis Witness
- ☐ Zoetis Vetscan
- ☐ Commercial laboratory
- ☐ Don't know
- ☐ Any available test
- ☐ Other:

8. Per your shelter's protocol in 2019, if a cat tested positive for **FeLV**, were any of the following tests routinely performed as a follow-up? **(check all that apply)**

- ☐ Follow-up tests were not routinely performed
- ☐ Any available test
- ☐ IFA test
- ☐ PCR test
- ☐ Antigen test at laboratory
- ☐ Not sure what types of tests were used
- ☐ Other:

9. Which of the following outcomes were routinely used for cats that tested positive for **FeLV** in 2019? **(check all that apply)**

- ☐ Cats could be adopted as a single cat or in a home with other FeLV+ cats
- ☐ Cats could be adopted regardless of the other cats in the home
- ☐ Cats could be transferred to rescue groups
- ☐ Cats could be transferred to sanctuaries
- ☐ Cats could be Trap-Neuter-Returned or Returned-to-Field
- ☐ Cats could be euthanized
- ☐ Other:

FIV Testing

Please respond with the most common protocols that were routinely followed in 2019, not exceptions or unusual circumstances.

10. In regards to routine **FIV** testing protocols in place in 2019 **(choose 1)**:

    ◯ **All cats** were tested – **PLEASE CONTINUE** to complete the following questions
    ◯ **Some cats** were tested - **PLEASE CONTINUE** to complete the following questions
    ◯ **No cats** were tested – **PLEASE SKIP** to the next section **Wrapping Up**

11. What types of cats were routinely tested for **FIV** in 2019? **(check at least one box in each row)**

**Type of Cat**	**No cats**	**Kittens (under 6 months old)**	**Adults (6 months & older)**	**N/A (shelter does not do this activity)**
Cats for adoption	☐	☐	☐	☐
Cats for transfer to other groups	☐	☐	☐	☐
Cats for Trap-Neuter-Return or Return-to Field	☐	☐	☐	☐
Cats housed individually in the shelter	☐	☐	☐	☐
Cats housed in group housing in the shelter	☐	☐	☐	☐
Cats that are sick or injured	☐	☐	☐	☐
Cats for whom the adopter/caregiver requests to be tested	☐	☐	☐	☐

12. If applicable and could not be answered in previous question, describe OTHER types of cats that were routinely tested for **FIV** in 2019:

13. What brand of test was routinely used for the initial **FIV** screening in 2019? **(check all that apply)**

    ☐ IDEXX SNAP
    ☐ Zoetis Witness
    ☐ Zoetis Vetscan
    ☐ Commercial laboratory
    ☐ Don’t know
    ☐ Any available test
    ☐ Other:

14. Per your shelter's protocol in 2019, if a cat tested positive for **FIV**, were any of the following tests routinely performed as a follow-up? **(check all that apply)**

- ☐ Follow-up tests were not routinely performed
- ☐ Western blot test
- ☐ PCR test
- ☐ Antibody test at laboratory
- ☐ Not sure what types of tests were used
- ☐ Other:

15. Which of the following outcomes were routinely used for cats that tested positive for **FIV** in 2019? **(check all that apply)**

- ☐ Cats could be adopted as a single cat or in a home with other FeLV+ cats
- ☐ Cats could be adopted regardless of the other cats in the home
- ☐ Cats could be transferred to rescue groups
- ☐ Cats could be transferred to sanctuaries
- ☐ Cats could be Trap-Neuter-Returned or Returned-to-Field
- ☐ Cats could be euthanized
- ☐ Other:

Wrapping Up

16. Submitter name:

17. Email:

18. Phone:

19. Position:
